# Supplementary material for: Investigation of anxiety levels and associated factor analysis in breast cancer patients undergoing chemotherapy with implanted venous access ports during the stable phase of disease
Source: PLoS One. 2025 Sep 25;20(9):e0332826. doi: 10.1371/journal.pone.0332826 (PMC12463248; doi:10.1371/journal.pone.0332826)
Supplement: Attachment — Submitted filename: Response.docx [file pone.0332826.s002.docx]

Dear Editors,

On behalf of all the contributing authors, I would like to express our sincere appreciations of your letter and reviewers’ constructive comments concerning our article. The following is our response to the comments:

1. We have included the start and end dates for accessing data for research purposes in the methods section of the article.

Thank you once again for your guidance and support!

Yours sincerely,

Purong Zhang
