# Supplementary material for: Investigation of anxiety levels and associated factor analysis in breast cancer patients undergoing chemotherapy with implanted venous access ports during the stable phase of disease
Source: PLoS One. 2025 Sep 25;20(9):e0332826. doi: 10.1371/journal.pone.0332826 (PMC12463248; doi:10.1371/journal.pone.0332826)
Supplement: Attachment — Submitted filename: Reviewers comments.docx [file pone.0332826.s003.docx]

**Reviewer’s Comments**

- **Sampling technique not well-detailed.**

**Suggestion:** Kindly clarify the sampling approach used to recruit the 920 eligible participants. For example, indicate whether a consecutive or convenience sampling strategy was employed. This will help readers evaluate the representativeness of the study population and potential biases.

- **No discussion of non-responders or missing data.**

**Suggestion:** Please include a brief discussion on non-responders or invalid questionnaires. Were there any systematic differences (e.g., demographic or clinical characteristics) between those who responded and those who did not? Even a statement acknowledging the limitation of not assessing this would be helpful.

- **No psychometric validation of SAS in this specific cohort (e.g., internal consistency):** Even though the SAS is a validated tool generally, its reliability can vary across populations, especially when an individual have a life threatening conditions like cancer. Without testing internal consistency in this population, it’s unclear whether the scale is truly measuring anxiety **accurately and consistently** in these patients.

**Suggestion:** Please consider reporting the internal consistency of the SAS scale in your sample (e.g., Cronbach’s alpha). Although SAS is widely validated, reporting this psychometric property will improve the reliability and generalizability of your anxiety findings in this unique cohort of breast cancer patients post-IVAP chemotherapy. If already available, this can be easily added to the Methods and Results sections. Or this could be mentioned in the limitation.

- **Improve Reporting of Variable Definitions.**

**Suggestion:** It would enhance the clarity and reproducibility of your study if you provide brief operational definitions or criteria used to assess subjective symptoms like “pulling sensation” or “foreign body sensation.” This will help standardize interpretation across different clinical contexts.
