# Supplementary material for: Investigation of anxiety levels and associated factor analysis in breast cancer patients undergoing chemotherapy with implanted venous access ports during the stable phase of disease
Source: PLoS One. 2025 Sep 25;20(9):e0332826. doi: 10.1371/journal.pone.0332826 (PMC12463248; doi:10.1371/journal.pone.0332826)
Supplement: Attachment — Submitted filename: Response to Reviewers.docx [file pone.0332826.s004.docx]

**Response**

**Reviewer 1：**

**• Sampling technique not well-detailed.
Suggestion: Kindly clarify the sampling approach used to recruit the 920 eligible participants. For example, indicate whether a consecutive or convenience sampling strategy was employed. This will help readers evaluate the representativeness of the study population and potential biases.**

Responses:

Thank you for the reviewers' suggestions. This study adopted a continuous sampling strategy. Based on the estimated sample size from the research statistics, patients who received implanted venous access ports and completed chemotherapy at the breast center of our hospital from January 1, 2019 to December 31, 2023 were included. According to the inclusion and exclusion criteria of the study, a total of 920 eligible patients were screened out. Non-respondents comprised 68 cases (Deceased patients (N=4); Patients diagnosed with other malignancies, severe systemic diseases (N=5); Breast cancer recurrence and metastasis during follow-up (N=21); Patients unable to provide valid responses due to insufficient literacy (N=16); Refuse to answer (N=19); Suffering from severe mental disorders or cognitive impairments (N=3)). A total of 76 questionnaires were excluded as invalid due to the following reasons: Patients lost to follow-up (N=17); Patients opted for IVAP maintenance at other hospitals (N=34); Patients opted for IVAP disease surveillance at other hospitals (N=14); Patients with incomplete data entry (resulting in unavailable clinical catheterization information) (N=11). Finally, 776 cases were included in the analysis questionnaire, with an effective response rate of 84.3%. This has been elaborated in the method section of the article, and the relevant tables of the patient screening flowchart have been supplemented in the research method section.

**Figure 1. Flowchart of Study Participant Selection**

**• No discussion of non-responders or missing data.
Suggestion: Please include a brief discussion on non-responders or invalid questionnaires. Were there any systematic differences (e.g., demographic or clinical characteristics) between those who responded and those who did not? Even a statement acknowledging the limitation of not assessing this would be helpful.**

Responses:

Thank you for the reviewers' suggestions. In this study, a total of 920 questionnaires were distributed. Of these, 776 valid questionnaires were retrieved. Non-respondents comprised 68 cases (Deceased patients (N=4); Patients diagnosed with other malignancies, severe systemic diseases (N=5); Breast cancer recurrence and metastasis during follow-up (N=21); Patients unable to provide valid responses due to insufficient literacy (N=16); Refuse to answer (N=19); Suffering from severe mental disorders or cognitive impairments (N=3)). A total of 76 questionnaires were excluded as invalid due to the following reasons: Patients lost to follow-up (N=17); Patients opted for IVAP maintenance at other hospitals (N=34); Patients opted for IVAP disease surveillance at other hospitals (N=14); Patients with incomplete data entry (resulting in unavailable clinical catheterization information) (N=11). Finally, a total of 144 cases were not included in the analysis.

During the statistical process, the researchers attempted to compare the demographic and clinical characteristics between the responders and the non-responders. Through the independent samples t-test and one-way analysis of variance (ANOVA) revealed no statistically significant differences, with all P＞0.05. However, among these 144 patients, the number of non-responders has limited access to relevant information, and there is still a possibility that the differences have not been identified. Therefore, a total of 776 valid questionnaires were obtained in the end, with an effective response rate of 84.3%.

Although the research team made every effort to verify the differences, due to the limitations of actual conditions, it was impossible to completely rule out the potential differences that might exist. This is indeed the limitation of sample inclusion in this study. Supplementary discussions have been made in the section on the limitations of the manuscript.

**• No psychometric validation of SAS in this specific cohort (e.g., internal consistency): Even though the SAS is a validated tool generally, its reliability can vary across populations, especially when an individual have a life threatening conditions like cancer. Without testing internal consistency in this population, it’s unclear whether the scale is truly measuring anxiety accurately and consistently in these patients.
Suggestion: Please consider reporting the internal consistency of the SAS scale in your sample (e.g., Cronbach’s alpha). Although SAS is widely validated, reporting this psychometric property will improve the reliability and generalizability of your anxiety findings in this unique cohort of breast cancer patients post-IVAP chemotherapy. If already available, this can be easily added to the Methods and Results sections. Or this could be mentioned in the limitation.**

Responses:

1.Thank you to the reviewers for your valuable comments！We have conducted a comprehensive literature search and have not yet found any reports on the internal consistency of the SAS Self-rating Anxiety Scale (such as Cronbach's α) in the population of breast cancer patients undergoing port of infusion (IVAP) chemotherapy. However, the SAS scale has been widely verified in the generalized breast cancer population, and its reliability and validity are good (usually α>0.80). In view of this situation, we have supplemented this issue in the the methods and results sections.

2.The Self-Rating Anxiety Scale (SAS) has been widely used to assess the anxiety levels of patients with breast cancer and other malignant tumors. In recent domestic and international studies, SAS has been employed to investigate the psychological states of breast cancer patients before surgery, during the perioperative period, and during chemotherapy. The SAS score during the perioperative period can serve as an important indicator of intervention effectiveness ^[1]^. Additionally, SAS has been utilized for breast cancer patients receiving chemotherapy through IVAP/PICC to evaluate the impact of different nursing models on anxiety ^[2]^. Regarding the psychometric properties of the scale, the Cronbach's α of the original Zung SAS ranges from 0.81 to 0.92, and domestic large-sample studies on breast cancer patients report an α coefficient of approximately 0.88 ^[3]^, suggesting good consistency among the items. The SAS consists of 20 items, is concise and easy to understand, and is a self-rating scale, which can avoid social desirability bias caused by face-to-face interviews. It is suitable for rapid screening of anxiety status during outpatient follow-up or chemotherapy breaks. Therefore, SAS is applicable for the investigation and research of breast cancer patients undergoing chemotherapy through port-a-cath.

References:

[1] Li J, Gao W, Yang Q, Cao F. Perceived stress, anxiety, and depression in treatment-naïve women with breast cancer: a case-control study. Psychooncology. 2021;30(2):231-239. doi:10.1002/pon.5555.
[2] Kim YH, Choi KS, Han K, Kim HW. A psychological intervention programme for patients with breast cancer under chemotherapy and at a high risk of depression: A randomised clinical trial. J Clin Nurs. 2018;27(3-4):572-581. doi:10.1111/jocn.13910.
[3] Hashemi SM, Rafiemanesh H, Aghamohammadi T, et al. Prevalence of anxiety among breast cancer patients: a systematic review and meta-analysis. Breast Cancer. 2020;27(2):166-178. doi:10.1007/s12282-019-01031-9.

**• Improve Reporting of Variable Definitions.
Suggestion: It would enhance the clarity and reproducibility of your study if you provide brief operational definitions or criteria used to assess subjective symptoms like “pulling sensation” or “foreign body sensation.” This will help standardize interpretation across different clinical contexts.**

Responses:

Thank you for your valuable suggestions. We fully recognize that clear variable definitions are crucial for the clarity and reproducibility of research. This has been detailed in the Methodology section.

For subjective symptoms such as "pulling sensation" and "foreign body sensation", the research team, by referring to existing literature and taking into account the actual situation of clinical patients, has made a detailed summary and elaboration of their definitions in the research methods section of the manuscript. In terms of "traction sensation", it is defined as a persistent or activity-induced tightness, pulling or constricting sensation perceived by breast cancer patients during the indwelling of an implantable venous port (IVAP) at the catheter site or in the surrounding tissues, often accompanied by local discomfort or limited movement. This subjective feeling may be related to the mechanical stimulation of the catheter on the surrounding tissues, fibrotic response or increased neural sensitivity, and it is an important factor affecting the patient's psychological state (such as anxiety) and quality of daily life. The "foreign body sensation" refers to the series of physiological and psychological reactions that an individual experiences when perceiving the existence of objects that do not belong to their normal tissues within or on their body surface. In medical contexts (such as patients with PICC or IVAP catheterization), foreign body sensation not only involves the physical perception of the catheter's presence but also the emotional, cognitive and behavioral responses it triggers.

We believe that these definitions are helpful in standardizing the interpretation of symptoms in different clinical contexts and improving the overall quality of research. Thank you again for your guidance.

References：

[1] Voog E, Campion L, du Rusquec P, et al. Totally implantable venous access ports: a prospective long-term study of early and late complications in adult patients with cancer. Support Care Cancer. 2018;26(1):81-89. doi:10.1007/s00520-017-3816-3.
[2] Ignatov A, Hoffman O, Smith B, et al. An 11-year retrospective study of totally implanted central venous access ports: complications and patient satisfaction. Eur J Surg Oncol. 2009;35(3):241-246. doi:10.1016/j.ejso.2008.01.020.
[3] Schiffer CA, Mangu PB, Wade JC, et al. Central venous catheter care for the patient with cancer: American Society of Clinical Oncology clinical practice guideline. J Clin Oncol. 2013;31(10):1357-1370. doi:10.1200/JCO.2012.45.5733.
[4] Voog E, Campion L, du Rusquec P, et al. Totally implantable venous access ports: a prospective long-term study of early and late complications in adult patients with cancer. Support Care Cancer. 2018;26(1):81-89. doi:10.1007/s00520-017-3816-3.

**Reviewer 2：**

**• Overall Summary of the Research**

**This study investigates the prevalence and associated factors of anxiety among female breast cancer patients in a stable disease phase who previously received chemotherapy through Implanted Venous Access Ports (IVAPs). Conducted at a single tertiary cancer hospital in China, the study involved 776 patients, using self-administered questionnaires and the Zung Self-Rating Anxiety Scale (SAS). Through univariate and multivariate regression analysis, the study identified several factors significantly associated with anxiety levels, including physical condition, wound dehiscence, sensations related to the IVAP (e.g., pulling or foreign body sensation), use of alternative venous access, and the sequence of surgery and chemotherapy.The manuscript addresses an important yet underexplored dimension of cancer survivorship care. While the research question is relevant and the sample size substantial, the study has some limitations regarding methodological structure, literature review, and manuscript structure that must be addressed prior to publication.**

Responses:

Sincerely thank the reviewers for their comments! We have revised and improved the structure of the methods we are studying, the literature review and the manuscript structure. You can view them in the revised manuscript section. Thank you sincerely again！

**MAJOR ISSUES：**

**• Study Design and Clarity- There is confusion about the timeline of the study.**

**The ethics approval is dated June 2024, while data were collected from patients treated between 2019 and 2023. It is unclear how retrospective consent was obtained. The methods need clear alignment of ethics, data access, and recruitment periods. The study is described as cross-sectional, but it includes retrospective data analysis. The authors should explicitly state this and address related limitations (e.g., recall bias).**

Responses:

Dear reviewers, thank you very much for your review of our research and your valuable suggestions. In response to your questions regarding the research timeline and methods, we hereby provide further explanations and clarifications.

Firstly, regarding the acquisition of retrospective consent: At the authors' affiliated hospital, ethical review requires successful completion of a preliminary assessment prior to granting full certification. The study protocol received preliminary IRB clearance on 30 May 2024 , with formal certification issued on 26 June 2024 (IRB Approval No: SCCHEC-02-2024-112). Participant recruitment was initiated strictly after obtaining the final IRB certification document. The research subjects were patients who had received chemotherapy for breast cancer and received IVAP implantation at the research team hospital from 2019 to 2023. The data collection was divided into two parts: one part was the collection of questionnaires, and the other part was the collection of relevant retrospective clinical medical records of the patients through the hospital's electronic information system and outpatient intravenous care system. Retroactive consent was obtained in written or oral form, and we have added this statement in the revised draft. The hospital where the research team is located is the largest specialized cancer hospital in the southwest of China, with a wide range of patient sources. Member Wen Min is a full-time follow-up management staff member. She regularly follows up with patients through phone calls, wechat, etc. Generally, patients will complete informed consent during regular electronic follow-ups, maintenance of outpatient intravenous access (IVAP), and outpatient disease re-examination cycles.

Secondly, regarding the research design: We confirm that this study adopted a cross-sectional design and included the part of retrospective data analysis. We will clearly elaborate on this design feature in the revised draft. Given the retrospective nature of the research data (i.e., reliance on participants' recollection of past exposure or outcome events), recall bias is an inherent methodological limitation of this design that is difficult to completely avoid. When discussing the limitations of the research, we will focus on elaborating on this, exploring its potential impact on the research results (such as the intensity or direction of exposure-outcome associations), as well as the efforts we have made during the research process to minimize its impact (for example: using validated structured questionnaires, controlling the interview environment, setting clear recall time anchors, etc.).

Finally, we would like to express our sincere gratitude once again for your review and guidance. We will carefully consider your suggestions and make corresponding modifications in the revised draft. We look forward to your valuable feedback again.

**• Instrument Validation- There is no report of cultural or linguistic validation of the Zung SAS for use in this specific patient population. Please clarify whether the version used has been validated in Chinese (if applicable), and provide a reference.**

Responses:

We thank the reviewers for their insightful comments. The Zung Self-Rating Anxiety Scale (SAS) used in this study is the officially validated Chinese version, which has undergone cultural and linguistic adaptation. Its reliability and validity have been established across multiple populations in China. The relevant validation studies and sources are outlined below:

**I. Reliability and Validity of the Chinese SAS Version**

•Reliability (Internal Consistency): Multiple studies indicate that the Chinese SAS demonstrates good internal consistency, with Cronbach's alpha coefficients around 0.85.

•Construct Validity: Exploratory Factor Analysis (EFA) and Confirmatory Factor Analysis (CFA) support a 3- or 4-factor structure for the SAS, encompassing dimensions such as anxious affect, autonomic symptoms, and somatic symptoms.

•Convergent Validity (Criterion Validity): The SAS shows significant correlations with the Beck Anxiety Inventory (BAI) (r = 0.34–0.71), confirming its effectiveness in measuring anxiety symptoms ^[1]^.

**II. Validation in Different Populations**

•Clinical Patients: Validated in patients with multimorbidity, showing good reliability (Cronbach’s α = 0.849) and construct validity ^[1]^.

•Civil Servants: A study of 8,590 civil servants established good reliability and validity (Cronbach’s α = 0.85). Reliability and validity further improved after removing Item 8 ("Fatigue"), with regional norms subsequently developed ^[2]^.

•University Students:The SAS exhibits sound psychometric properties in Chinese student populations, with anxiety detection rates consistent with clinical standards ^[3]^.

**III. Cultural Adaptation and Revision**

• Some studies recommend modifying or deleting specific items (e.g., Item 8 "Fatigue") to enhance the scale’s applicability ^[1]^.

•The Chinese norm typically uses a cut-off score of ≥50 (indicating mild anxiety), which differs slightly from international standards.

References

[1] Fu Y, Wu J, Zhao B, Lai C, Xue E, Wang D, Wang M, Tang L, Shao J. Development of a Chinese version of the Stress Adaption Scale and the assessment of its reliability and validity among Chinese patients with multimorbidity. Zhejiang da xue xue bao. Yi xue ban= Journal of Zhejiang University. Medical Sciences. 2023 Jun 1;52(3):361-70.
[2] Qiao Y, Wang J, Zhang C. Reliability, validity test and norm construction of the self-rating anxiety scale in civil servants.Adv Psychol. 2022;12(6):10. doi:10.12677/AP.2022.126270.
[3] Zhao X, Hu T, Qiao G, et al. Psychometric properties of the smartphone distraction scale in Chinese college students: validity, reliability and influencing factors[J]. Frontiers in psychiatry, 2022, 13: 859640.

**• Confounding and Covariates- The regression model does not appear to adjust for potential confounders such as age, cancer stage, treatment type, or socioeconomic status, which could influence anxiety. These should be included or justified for exclusion. It is unclear how multicollinearity was checked in the regression model, especially given several closely related variables (e.g., symptoms from IVAP, physical condition, etc.).**

Responses:

Thank you for pointing out these important issues in your review. Thank you for your valuable comments during the review. The potential confounding factors you pointed out, such as age, cancer stage, type of treatment and socioeconomic status, do indeed have a crucial impact on anxiety levels. In response to your concerns, we make the following explanation: During the initial analysis stage, we conducted a systematic univariate analysis of the above variables (age, cancer stage, treatment type, socioeconomic status). The results showed that the inter-group differences in the anxiety score (SAS) of these variables did not reach statistical significance (P > 0.05). This indicates that in the current research sample, these factors do not show a significant association with anxiety levels, so their potential impact as confounding variables may be limited. Secondly, the construction of a multi-factor regression model needs to balance the number of variables and statistical power. Incorporating too many non-significant variables may lead to overfitting of the model, reducing estimation accuracy (such as an increase in standard errors) or masking the true effects of core variables. Therefore, based on this information, we decided not to include these variables in the multivariate analysis.

Furthermore, regarding the issue of multicollinearity, the research team has conducted a thorough examination in the regression model, especially taking into account the conditions of multiple related variables such as IVAP symptoms and physical conditions. We will address this issue in the revised draft and introduce the process of collinearity analysis before the regression model to prove the rationality of the variables we included in the model and enhance the reliability of our analysis.

**• Literature Review and Theoretical Framing- The introduction and discussion underutilise international literature. For a PLOS ONE audience, broader referencing beyond national studies (e.g., NCCN or ESMO psycho-oncology guidelines) would strengthen the scientific context. Some references are missing or shown as placeholders (e.g., “Error! Reference source not found.”). Please revise for completeness and accuracy.**

Responses:

Thank you very much for your valuable suggestions on the literature review and theoretical framework of this research. The issues you pointed out regarding the insufficient utilization of international literature and the problems with references are of great guiding significance for us to improve our research. We fully agree and will carefully revise them.

In response to the issue of insufficient citations of international literature, we will systematically supplement high-quality literature related to this research topic in international authoritative journals in the past five years, especially the relevant achievements published in PLOS ONE, in order to better meet the academic needs of journal readers. Meanwhile, we will focus on referring to the latest psycho-oncology guidelines released by NCCN (National Comprehensive Cancer Network of the United States), ESMO (European Society for Medical Oncology), etc., to compare and analyze the similarities and differences between international and domestic research, further expand the scientific context of the research, and enhance the international relevance of the research conclusions. The following are the additional citations we have added. For details, please refer to the introduction and discussion sections in the revised draft.

Regarding missing references or placeholders (such as "Error! Reference source not found.") , these might be our oversights in the initial draft writing and format proofreading. When making revisions, we will conduct a comprehensive review of all references in the text, supplement and improve missing citations, correct format errors, and ensure that all references are accurate, complete and standardized.

Thank you again for your meticulous guidance. We will strictly follow the suggestions to make revisions and improvements, and strive to enhance the academic quality of our research.

Added references:

[1] Gradishar WJ, Moran MS, Abraham J, et al. Breast Cancer, Version 3.2024, NCCN Clinical Practice Guidelines in Oncology. J Natl Compr Canc Netw. 2024;22(5):331-357. doi:10.6004/jnccn.2024.0035.
[2] Loibl S, André F, Bachelot T, et al. Early breast cancer: ESMO Clinical Practice Guideline for diagnosis, treatment and follow-up. Ann Oncol. 2024;35(2):159-182. doi:10.1016/j.annonc.2023.11.016.
[3] Liu B, Wu Z, Lin C, Li L, Kuang X. Applicability of TIVAP versus PICC in non-hematological malignancies patients: A meta-analysis and systematic review. PLoS One. 2021;16(8):e0255473. Published 2021 Aug 3. doi:10.1371/journal.pone.0255473.
[4] Berger AM, Abernethy AP, Atkinson A, et al. NCCN Clinical Practice Guidelines Cancer-related fatigue. J Natl Compr Canc Netw. 2010;8(8):904-931. doi:10.6004/jnccn.2010.0067.
[5] Sanft T, Day A, Ansbaugh S, et al. NCCN Guidelines® Insights: Survivorship, Version 1.2023. J Natl Compr Canc Netw. 2023;21(8):792-803. doi:10.6004/jnccn.2023.0041.

**• Language and Grammar- The manuscript requires extensive English language editing to improve clarity and fluency. Grammatical errors, awkward phrasing, and overly literal translations (e.g., "bad physical condition") detract from the overall quality.**

Responses:

Thank you very much for pointing out the problems in the language and grammar of this article. The suggestions you put forward regarding clarity, fluency and accuracy of expression are crucial for improving the quality of the manuscript, and we fully agree with this.

In response to the issue that requires comprehensive revision of the English expression, we will hire professional native speakers with experience in editing papers in the medical or related fields to polish the entire text, focusing on correcting grammatical errors and adjusting stiff expressions (such as optimizing "poor physical condition" to "poor general health status" and similar expressions that are more in line with academic norms). Ensure that the text is smooth, natural, professional and accurate. Meanwhile, we will check the entire text sentence by sentence to avoid semantic deviations caused by literal translation, so that the research content can be clearly and accurately conveyed to readers.

We are well aware that the standardization of language expression is an important manifestation of the quality of academic papers. We will complete the revision with a rigorous attitude to ensure that it meets the publication requirements of the journal. Thank you again for your detailed guidance.

**MINOR ISSUES：**

**• Tables and Figures- Tables are dense and could benefit from clearer headings, footnotes, and consistent formatting. Consider highlighting statistically significant results for easier interpretation. A flow diagram of patient inclusion/exclusion and a figure visualising anxiety severity distribution would enhance clarity.**

Responses:

Thank you for your feedback on the tables and charts in our manuscript. We have noticed that the table content is rather dense and will consider improving the table header, annotations and format to enhance clarity and maintain consistency. At the same time, we will also consider marking the statistically significant results to make them easier to interpret. In addition, we will create a flowchart that includes the inclusion/exclusion criteria for patients, as well as a chart showing the distribution of anxiety severity, to enhance clarity and better visualize the data presented in the manuscript.

Thank you sincerely again for your suggestions. We will make necessary adjustments to improve the presentation of tables and charts in the manuscript. Thank you for your guidance.

**Figure 2. Distribution of Anxiety Severity in Patients**

|  | Figure 1. Distribution of Anxiety Severity in Patients |
| --- | --- |
| mild anxiety | 40.21% |
| moderate anxiety | 15.98% |
| severe anxiety | 2.32% |

**• Ethical Clarity- The ethics section mentions both “preliminary review” and “final ethics approval” dated after data collection began. Please clarify when ethical approval was granted in relation to participant recruitment.**

Responses:

Thank you for your feedback on the clarity of the ethical issues in our manuscript. We understand the importance of providing clear information on the time of ethical approval in participant recruitment.

We will revise the ethics statement to clarify the institutional review board (IRB) approval process. At the authors' affiliated hospital, ethical review requires successful completion of a preliminary assessment prior to granting full certification. The study protocol received preliminary IRB clearance on 30 May 2024 , with formal certification issued on 26 June 2024 (IRB Approval No: SCCHEC-02-2024-112). Participant recruitment was initiated strictly after obtaining the final IRB certification document. This amendment ensures full compliance with ICMJE guidelines and enhances methodological transparency regarding ethical governance.

Thank you for your attention to this matter. We will make necessary adjustments to enhance the ethical transparency of the research. Thank you for your valuable suggestions.

**• Limitations- The limitations section is quite brief. It should discuss:
a) Single-centre recruitment
b) Use of self-reported outcomes
c) Lack of clinical anxiety diagnosis (vs. screening scale)
d) No data on supportive care interventions or prior psychiatric history**

Responses:

We sincerely appreciate your constructive suggestions regarding the limitations section of our manuscript. Acknowledging that the current description is relatively concise, we will expand this section to incorporate the following key methodological considerations:

a) Limited generalizability: Single-center recruitment with restricted sample size may introduce homogeneity in demographics, disease profiles, and institutional practices, constraining external validity.

b) Measurement biases: Self-reported anxiety (via SAS) risks response/recall biases; Lack of structured clinical interviews (e.g., SCID) prevents diagnostic differentiation; Unvalidated SAS psychometrics in port-using chemotherapy cohorts

c) Uncontrolled confounders: Inadequate accounting for psychosocial interventions and pre-existing mental health conditions.

d) Temporal constraints: Short post-chemotherapy assessment window precludes evaluation of long-term psychological effects. Unavailability of data on supportive care interventions or pre-existing psychiatric conditions.

The revised manuscript now includes a comprehensive discussion of these limitations, addressing their potential impact on result interpretation and generalizability. We thank you for these insightful recommendations, which significantly strengthen the scholarly rigor of our work.

**• Data Availability- While the manuscript states that all data are available in supplementary files, ensure that datasets are in a machine-readable format and include a DOI if applicable, in line with PLOS data policies.**

Responses:

Thank you very much for your valuable suggestions regarding the availability of data for this study. The requirements you highlighted concerning data format and DOI are essential for us to adhere strictly to PLOS data policy and enhance the reproducibility of our research. We fully agree with these points and will implement them diligently. To improve data availability, we will undertake the following specific measures:

Firstly, we will conduct a comprehensive review to ensure they are converted into machine-readable formats (such as CSV, TSV, or Excel) that comply with PLOS requirements. This will facilitate direct access and secondary analysis by other researchers. Secondly, for datasets eligible for DOI assignment, we will archive them through recognized data storage platforms (such as Figshare or Dryad) and apply for unique DOIs. Once obtained, we will promptly indicate these DOIs in the text to ensure permanent traceability of the data. Finally, if necessary, we will detail methods for obtaining the data, including information on storage platforms, formats used, and DOI details (if applicable), thereby adhering strictly to journal norms regarding data sharing.

We are committed to ensuring that all supplementary materials related to the data are complete and standardized in accordance with PLOS's requirements for transparency and accessibility. We have appended the supplementary supporting information files to the end of the revised manuscript and have also uploaded them to Figshare(DOI:10.6084/m9.figshare.29959559). Thank you once again for your insightful guidance; we aim to enhance academic rigor through these practical modifications.
